# Sporadic Pleuro-pneumonia1Read before the Western Ontario Veterinary Medical Association.

**Published:** 1896-06

**Authors:** F. A. Thomas

**Affiliations:** Tava, Ontario


### SPORADIC PLEURO-PNEUMONIA.[1]

By F. A. THOMAS, V.S.,
TAVA, ONTARIO.

This disease possesses much interest for the veterinary profession, and is the cause of much concern to the agriculturist. Its likeness to the contagious type has led to an embargo being placed on our cattle entering the British market. Sporadic pleuro-pneumonia in its symptoms very much resembles the dreaded fatal contagious form. The reports of the English veterinary inspectors prove that they have met with only the sporadic form. The lungs and the reflexed portion of the pleura have been affected the same as we find on post-mortem examination in the local disease. Had it been the contagious form the symptoms would have been much more aggravated than in the animals condemned and slaughtered. The surroundings were so favorable that, instead of one or two animals being affected, many of the cargo would have been attacked by the disease; being stalled so closely together, and so many in number, the air which they breathed must have become more or less vitiated, which would greatly favor the spread of any contagious disease.

On the other hand, everything was favorable to the development of the local or sporadic form of lung trouble, a great majority of the animals going directly from warm stalls in stone stables to the seaport, and in all probability contracting the disease before being put aboard ship. The currents of air while in transit in the cars, especially in a night run, are the most common cause of the disease as we meet it in our practice; and the post-mortem examinations I have found correspond very closely with those described in the contagious form of pleuro-pneumonia and also with those described by the veterinary surgeons who examined the lungs of the animals found affected. The chief differential diagnostic symptoms which I have met in

[1] Read before the Western Ontario Veterinary Medical Association.

sporadic pleuro-pneumonia from those described in the contagious form have been the yellowish discharge from the eyes and nostrils and the tendency to purgation on a fatal termination of the disease.

Many cases arise from allowing a draught or cold current of air to pass through the stable until the animals become chilled, or turning them out of a warm stable to drink and letting them remain out in the cold and snow, as many farmers say, for exercise, too closely crowding in a stable with poor ventilation, and the air becoming vitiated.

The symptoms of the disease are not very well marked at the start. It is generally ushered in with chills or rigors, with a soft pulse, about 60. The breathing in a short time becomes quickened and shallow. On auscultation a crackling sound is heard over the part affected; ears and horns become alternately hot and cold as the disease advances; rumination ceases, bowels become costive, urine scanty and high colored. Temperature 104° or 105°, with considerable coughing in spells. The lungs become generally affected, and in four or five days more or less effusion can be detected in the thoracic cavity; mucous discharge from the nostrils. When standing the animals keep the elbows out from the body, and in lying keep well up on the sternum. Temperature ranges between 105° and 106°. Pulse 75 to 80. The breathing becomes painful, causing a groan on expiration, which is increased by percussion. Bowels become obstinately constipated. The animal may live for ten to fourteen days, when death closes the scene.

The treatment is generally successful when called in time. Remove the animal to a comfortable loose box, if possible, away from other animals, and protected from all draughts. Give 20 minims of aconite and 2 ounces of nitrate of potassium every four hours. Sew a large blanket around the animal, and wet it with hot water every ten minutes for six or eight hours, then rub dry; apply a mustard application over the thoracic walls. Clothe comfortably according to the season. Shut off all coarse diet, as the coarse feed passes into the rumen, and fermentation taking place causes the animal considerable distress by pressing on the diaphragm. Keep on a sloppy diet with as much linseed-tea as possible, with frequent injections to regulate the bowels. As the animal improves drop off in the medicinal treatment.

This being the disease which has without a doubt caused the trouble in our cattle trade, our only hope for its removal is to have our government make a strenuous effort in the proper direction, by appointing a commission to thoroughly investigate it in every section where it has been prevalent, and with the united aid of the veterinary profession I feel sure we can satisfy the English authorities that contagious pleuro-pneumonia does not exist within the Dominion of Canada.

---

Dr. R. A. Denis, Genung, N. Y., reports the following interesting anomaly: A brood-sow give birth to eight pigs, two males, both perfect; six females, none of which had any vulva. They were four weeks old when seen. One was killed for the purpose of examination, when a fistulous opening from the vagina to the rectum was discovered, and urine and feces were expelled from the anus. All the pigs appeared to be in good health.

The antitetanic serum prepared by the Pasteur Laboratory of Paris is not claimed to be a specific cure for tetanus, but to have its chief virtue and use in those cases where premonitory symptoms of tetanus may supervene, or where they might be anticipated; for instance, in certain stables where a number of cases may have occurred at intervals following nail or slight wounds of the extremities. Ten cubic centimetres are recommended as a sufficient dose for immunizing, which should be repeated in a week when any doubt exists. The thorough antiseptic care of wounds is the greatest safeguard in all cases.

A dog in southern New Jersey is reported as contracting whooping-cough from a child and subsequently dying in one of the coughing spells.

Röntgen rays have been used at the State University at Columbia, Mo., for the destruction of diphtheria germs in inoculated guinea-pigs, with successful results after an exposure of three hours. An inoculated pig not so exposed died in twenty-eight hours.

Fourteen quarts of milk in twenty-four hours are said to have been taken from a mare at the expiration of the period of suckling her colt.

